# INVOLVING PERSONS LIVING WITH DEMENTIA AS KEY STAKEHOLDERS: RESEARCH “WITH” RATHER THAN RESEARCH “ON”

**DOI:** 10.1093/geroni/igac059.1872

**Published:** 2022-12-20

**Authors:** Ashlee Cordell, Christa Wilk, Silvia Orsulic-Jeras, Sara Powers, Farida Ejaz, Beth Sanders

**Affiliations:** Benjamin Rose Institute on Aging, Cleveland, Ohio, United States; Benjamin Rose Institute on Aging, Cleveland, Ohio, United States; Benjamin Rose Institute on Aging, Cleveland, Ohio, United States; The Benjamin Rose Institute on Aging, Cleveland, Ohio, United States; Benjamin Rose Institute on Aging, Cleveland, Ohio, United States; LifeBio, MARYSVILLE, Ohio, United States

## Abstract

Involving persons living with dementia (PLWD) as members of the research team is an important step in establishing an environment of conducting research “with” as opposed to research “on”. Emerging research is also capturing PLWDs' lived experiences to inform product development. This presentation highlights the importance of conducting product development research with PLWD as key stakeholders and the valuable insight they provide to researchers and developers. Five community-dwelling PLWD with varying diagnoses (60% mild cognitive impairment; 20% Alzheimer’s Disease; 20% mixed dementia) and tMMSE scores ranging from 22-26 (Mean=25) participated in two rounds (9 months apart) of 60-minute-long virtual focus groups via Zoom to test multiple versions of LifeBio MemoryTM: an app-based product designed for PLWD which utilizes advanced technology to improve an existing life story intervention. In Time 1, PLWD provided feedback on prototypes of the app and associated training that was used to inform the final product. In Time 2, participants reviewed the final product. A thematic analysis was conducted at each time point to inform further development of the app. Findings determined the following themes: 1) life stories can help with providing better person-centered care, 2) potentially sensitive topics require more training, 3) simple and clear instructions are vital for success. Topics further discussed in this presentation will include PLWDs': 1) thoughts on the concept of LifeBio and its adaptation, 2) comfort with answering life story interview questions, 3) opinions on dementia-friendly materials, and 4) reactions to the use of app-based technology.

